# Effect of *Lactobacillus acidophilus* Fermented Broths Enriched with *Eruca sativa* Seed Extracts on Intestinal Barrier and Inflammation in a Co-Culture System of an Enterohemorrhagic *Escherichia coli* and Human Intestinal Cells

**DOI:** 10.3390/nu12103064

**Published:** 2020-10-07

**Authors:** Francesca Bonvicini, Eleonora Pagnotta, Angela Punzo, Donato Calabria, Patrizia Simoni, Mara Mirasoli, Nadia Passerini, Serena Bertoni, Luisa Ugolini, Luca Lazzeri, Giovanna Angela Gentilomi, Cristiana Caliceti, Aldo Roda

**Affiliations:** 1 Department of Pharmacy and Biotechnology—FABIT, University of Bologna, 40126 Bologna, Italy; francesca.bonvicini4@unibo.it (F.B.); nadia.passerini@unibo.it (N.P.); serena.bertoni4@unibo.it (S.B.); giovanna.gentilomi@unibo.it (G.A.G.); 2 CREA-Council for Agricultural Research and Economics, Research Centre for Cereal and Industrial Crops, via di Corticella 133, 40128 Bologna, Italy; eleonora.pagnotta@crea.gov.it (E.P.); luisa.ugolini@crea.gov.it (L.U.); luca.lazzeri@crea.gov.it (L.L.); 3 Department of Chemistry “Giacomo Ciamician”, University of Bologna, 40126 Bologna, Italy; angela.punzo2@unibo.it (A.P.); donato.calabria2@unibo.it (D.C.); mara.mirasoli@unibo.it (M.M.); aldo.roda@unibo.it (A.R.); 4 Department of Medical and Surgical Sciences—DIMEC, University of Bologna, 40126 Bologna, Italy; patrizia.simoni@unibo.it; 5 Department of Biomedical and Neuromotor Sciences—DIBINEM, University of Bologna, 40126 Bologna, Italy; 6 Istituto Nazionale Biosistemi e Biostrutture—INBB, 00136 Rome, Italy

**Keywords:** lactic acid bacteria, fermentation, *Brassicaceae*, glucosinolates, intestinal inflammation, gut barrier, enterohemorrhagic *Escherichia coli*

## Abstract

Lactic acid bacteria (LAB) “fermentates” confer a beneficial effect on intestinal function. However, the ability of new fermentations to improve LAB broth activity in preventing pathogen-induced intestinal inflammation and barrier dysfunction has not yet been studied. The objective of this study was to determine if broths of LAB fermented with *Eruca sativa* or *Barbarea verna* seed extracts prevent gut barrier dysfunction and interleukin-8 (CXCL8) release in vitro in human intestinal Caco-2 cells infected with enterohemorrhagic *Escherichia coli* (EHEC) O157:H7. LAB broths were assayed for their effects on EHEC growth and on Caco-2 viability; thereafter, their biological properties were analysed in a co-culture system consisting of EHEC and Caco-2 cells. Caco-2 cells infected with EHEC significantly increased CXCL8 release, and decreased Trans-Epithelial Electrical Resistance (TEER), a barrier-integrity marker. Notably, when Caco-2 cells were treated with LAB broth enriched with *E. sativa* seed extract and thereafter infected, both CXCL8 expression and epithelial dysfunction reduced compared to in untreated cells. These results underline the beneficial effect of broths from LAB fermented with *E. sativa* seed extracts in gut barrier and inflammation after EHEC infection and reveal that these LAB broths can be used as functional bioactive compounds to regulate intestinal function.

## 1. Introduction

The use of microbial fermenters has been instrumental in making a large range of foods, popular around the world. Numerous studies have evidenced the effect of yogurt consumption on chronic disease risk biomarkers in adults [1], while relatively few human clinical studies on the effects of fermented vegetables on health outcomes have been described in literature [2]. Sauerkraut, kimchi and cortido are all products of fermented *Brassicaceae*, and their recipes originated in North Europe, South Korea and Central America respectively [3].

Lactobacilli fermented cruciferous vegetables are often rich in other functional ingredients such as garlic, ginger, and red pepper powder which are themselves very rich in vitamins, minerals, and dietary fibres [4,5]. Although fermentation is considered to be a major factor responsible for the favourable effects of Brassicaceae vegetables in improving overall food quality, few evidences supporting this concept have yet been reported. Fermentation can be regulated in order to enhance the content of vitamin B12 [6], or vitamin C [7], depending on the starting fresh vegetable, the bacterial inoculum, and the presence of other ingredients. More recently Odongo et al. studied the differences in composition of secondary plant metabolites in raw, fermented, and cooked *Brassica carinata* leaves, highlighting a sharp decrease in glucosinolate (GSL), a unique class of plant secondary metabolites [8], a structure-dependent degradation of phenolic compounds in fermented products in comparison to fresh leaves, and a slight increase in isothiocyanates, i.e. GSL hydrolysis products, in comparison to both raw and cooked vegetable materials [9].

The role of non-traditional therapies such us the consumption of fermented dairy or non-milk-derived foods for the management of intestinal inflammatory diseases is a subject of current interest [2]. Recent studies in cellular and animal models focus on the effects of probiotics (live bacteria that offer benefits to the host) on various disease states, including inflammatory bowel diseases (IBD) [10].

Lactic acid bacteria (LAB), such as Lactobacillus species, are probiotic microorganisms known to have protective effects against a variety of pathogenic infections in the gastrointestinal systems of humans and animals [11,12]. As an example, *Lactobacillus rhamnosus* GG (LGG) bacteria are reported to confer beneficial effects on epithelial intestinal cells, including antagonizing infections and reducing overt proinflammatory responses [13].

There is an increased interest in ways to alter the composition and function of the gut microbiota without the introduction of exogenous live microbes [14]. Several studies suggest that the effect caused by different LAB is broth-specific and reveal that broths can be used as functional bioactive compounds, for example in preventing food contamination [15]; however, LAB are widely used as starter cultures for the production of diverse fermented foods but, to the best of our knowledge, the potential use of their fermented broth in the food industry has not been extensively studied.

In vitro studies highlighted the potential influence of well-known polyphenols on the intestinal microbiota commonly present in the human gastrointestinal tract, by influencing the adhesion of two representative gut bacteria, *Salmonella typhimurium* (a Gram-negative pathogen) and LGG (a Gram positive probiotic) [16]. The growth of different lactobacilli has been previously investigated using different polyphenol matrices [17]. Recently, the probiotic and immunomodulatory properties of different lactobacilli isolated from fermented Brassicaceae such as radish or Chinese cabbage (*Brassica rapa* L. ssp. *Pekinensis*), known as kimchi, were examined. Isolated lactobacilli showed a broad-spectrum antimicrobial activity, and a significant adherence to the intestinal epithelial cell line Caco-2 in comparison with the probiotic LGG [18] and improved clinical signs via immune modulation in a dextran sodium sulphate-induced colitis mouse model [19].

Moreover, the behaviour of three Lactobacillus strains, grown in the presence of previously chopped *Eruca sativa* Miller leaves, was recently investigated [20]. Rocket leaves, as with all Brassicaceae tissues, contain GSLs, which are hydrolysed when they come in contact with the plant or bacterial enzyme myrosinase, leading to the formation of several intermediate products, such as isothiocyanates [8] The presence of these molecules in the growth mediums of Lactobacilli affect the antioxidant activity of the medium, the specific antioxidant power of lactobacilli, and their antimicrobial activities [20].

However, to our knowledge, there is no literature reporting the effect of Brassicaceae seed extracts on lactobacilli fermented broths’ efficacy in preventing pathogen-induced intestinal barrier dysfunction and inflammation.

In this study, the ability to improve the epithelial intestinal barrier function, the regulation of pro- and anti-inflammatory gene expression, and the potential role in altering infectious processes by lactobacilli broths enriched or not with *Brassicaceae* seed extracts were evaluated in vitro in a Caco-2 cell monolayer system. In particular, *E. sativa,* synonym of *E. vesicaria subsp. sativa (Miller) Thell.* which is the only taxon of *Eruca vesicaria (L.) Cav.* cultivated worldwide, and *Barbarea verna* (Mill.) Asch seeds were chosen for their glucosinolate (GSL) content and profile. *E. sativa* and *B. verna* seeds contain a high amount of 4-methylthiobutyl GSL (glucoerucin) and 2-phenylethyl GSL (gluconasturtiin) respectively [21]. Both GSLs form isothiocyanates (ITCs) upon hydrolysis caused by the plant endogenous myrosinase enzyme, under physiological conditions, upon tissue disruption. 4-methylthiobutyl GSL is the precursor of 4-methylthiobutyl ITC, or ericin, which is considered as a flavoring agent or adjuvant for foods by the Food and Drug Administration (FDA) and the Center for Food Safety and Applied Nutrition (CFSAN). Erucin is also known as an emerging bioactive molecule involved in cancer prevention [22], and as an H_2_S donor with antihypertensive and anti-hyperalgesic activities [23]. Moreover, this molecule has well-known in vitro antioxidant and antidiabetic activities [24]. Gluconasturtiin is the precursor of 2-phenylethyl ITC; it is considered a flavoring agent or adjuvant in food as erucin; nevertheless, its effects on health are better known in comparison to those which are emerging for erucin. Indeed 2-phenylethyl ITC has been shown to target signaling pathways that are involved in the proliferation and survival of several cancer cell lines [25,26], and antimicrobial activity against harmful intestinal bacteria such as *Clostridium difficile*, *Clostridium perfringens*, enterohemorrhagic *Escherichia coli* (EHEC) as well as Shiga toxin production, while it showed no effect on beneficial bifidobacteria and lactobacilli growth [27,28]. EHEC strains of serotypes O157:H7 are foodborne pathogens related to large global outbreaks and responsible for human diseases ranging from uncomplicated diarrhea to bloody diarrhea or hemorrhagic colitis (HC) and life-threatening sequelae, such as thrombocytopenic purpura and hemolytic uremic syndrome [29]. The mechanisms of the antimicrobial activity of ITCs are still not well understood. The high electrophilic ITC group could react with amine, thiol or hydroxyl groups; thus, it may influence activities and functions of molecular targets in bacterial cells. Current knowledge suggests that aromatic ITCs seem to be more effective than aliphatic ones against various bacteria, but there are no general rules [30]. Nevertheless, most reports are limited to the determination of minimum inhibitory concentration (MIC), which may vary depending on the method used, but showed a dose dependent activity against pathogen bacteria which could evidence selective inhibitory activity against harmful intestinal bacteria in comparison to beneficial bifidobacteria and lactobacilli [31].

The aim of the present study was to exploit the ability of *Lactobacillus acidophilus* fermented broths enriched with *E. sativa* or *B. verna* seed extracts to prevent the intestinal barrier dysfunction and inflammatory response caused by the human pathogen EHEC. These biological properties were assayed on the human Caco-2 cell line, a well-recognized in vitro model of intestinal epithelium [32], after having assessed the safety of the broth solutions in terms of viability and the modelling of a “prêt a porter” human intestinal infection system.

## 2. Materials and Methods

### 2.1. Chemicals

Phosphate-buffered saline (PBS) tabs (a 137 mM NaCl, 2.7 mM KCl and 10 mM phosphate buffer solution, pH 7.4), trypsin-EDTA and 100X antibiotic solution (10,000 U/mL penicillin and 10 mg/mL streptomycin), Lipopolysaccharides (LPS), Bovine Serum Albumin (BSA), and Triton X-100 were purchased from Sigma-Aldrich (St Louis, MO, USA).

Dulbecco’s Modified Eagle Medium (DMEM) high glucose Non-Essential Amino Acids solution (NEAA) and Fetal Bovine Serum (FBS) were purchased from Microgem (Naples, Italy). WST8 (2-(2-methoxy-4-nitrophenyl)-3-(4-nitrophenyl)-5-(2,4-disulfophenyl)-2H-tetrazolium, monosodium salt) was purchased from Dojindo Molecular Technologies (Japan). RNeasy Mini Kit was from QIAGEN (Hilden, Germany). Primers for RT-PCR were purchased from IDT (Coralville, IA, USA). SuperScript^®^ III First-Strand Synthesis SuperMix and EXPRESS SYBR^®^ GreenER™ qPCR Super-Mix were purchased from Life Technologies (Carlsbad, CA, USA). All the other chemicals and solvents were of the highest analytical grade.

Rabbit polyclonal antibody to zona occludens-1 (ZO-1) was purchased from Biorbyt (Cambridge, UK), and goat anti-rabbit IgG DyLight488 conjugate from ImmunoReagents Inc. (Raleigh, NC, USA). Human CXCL8 ELISA kit was purchased from Elabscience (USA), cat.n. E-EL-H6008.

Columbia agar with 5% sheep blood, Mueller-Hinton (MH) broth, and de Man, Rogosa and Sharpe (MRS) broth were purchased from Oxoid (Basingstoke, Hampshire, UK).

The standard of allyl glucosinolate was isolated as previously reported [33] at HPLC purity > 99 % and stored at −20 °C until required. Sulfatase from *Helix pomatia* (Sigma Aldrich, St. Louis, MO, USA) was purified according to ISO 9167-1:1992/Amd 1:2013 [34] and stored at −20 °C until required.

### 2.2. Plant Materials and Extracts

*E. sativa* var. NEMAT and *B. verna* were from the Brassicaceae collection at the Council for Agricultural Research and Economics (CREA-CI) Bologna, Italy [35], and were grown during the season 2014–2015 and 2016 respectively, adopting a minimum agronomical input approach [36]. The cultivation was carried out at the CREA experimental farm located in Budrio (Bologna) in the Po Valley area (Emilia Romagna region, 44°32′00″ N; 11°29′33″ E, altitude 28 m a.s.l.). After harvesting, *E. sativa* and *B. Verna* seeds were threshed and air-dried to reduce the high residual moisture content. Seeds were defatted using a small seed continuous crusher machine (Bracco Company model Elle. Gi type 0.90) with a procedure during which temperature was maintained at a maximum of 70 °C. *E. sativa* and *B. Verna* defatted seed meals (DSMs) were characterized for residue oil content by standard automated continuous extraction, following the Twisselmann principle, by using a E-816 ECE (Economic Continuous Extraction) extraction unit (BÜCHI Labortechnik AG, Switzerland), and hexane as solvent. The extracts were prepared starting from 30 g DSM in 300 mL of 30% ethanol divided in 12 Teflon vessels and extraction was performed using the Microwave Extraction System MARS (CEM corporation), setting at 400 W as maximum power, heating ramp up to 80 °C in 3 min, and maintaining at 80 °C for further 10 min. Obtained extracts were sonicated for 30 min in a Sonica Sweep System (Soltec) bath at 40 kHz and then centrifuged and filtered, before being collected and stocked in Pyrex bottles at −20 °C for 48 h. Finally, they were immediately filtered in 0.45 µm polytetrafluorethylene (PTFE) membranes and concentrated using vacuum rotavapor (40 °C) in order to gain a volume reduction of approximately 15 times. GSL concentration of filtered extracts (cellulose acetate 0.45 μm syringe filter) was determined by HPLC–UV analysis of desulphated-GSL [37]. The desulphated GSLs were detected by monitoring their absorbance at 229 nm and initially identified with respect to their retention times and UV spectra according to our library [21,22,23,24,25,26,27,28,29,30,31,32,33,34,35,36,37,38]. The amounts were estimated using allyl GSL as internal standard; the response factor for desulphated glucoerucin, glucoraphanin GSL, and gluconasturtiin were according to [33].

### 2.3. Probiotic Bacteria Strain and Culture Conditions

*Lactobacillus acidophilus* (SD5212) broths were supplied by Incos S.r.l. (Bologna, Italy). Briefly, bacteria were anaerobically grown at 37 °C in MRS broth in the presence and the absence of 2 mM total GSLs of which 96% was represented by glucoerucin and 4% by glucoraphanin, from *E. sativa* DSM extracts (2 mL aqueous extract in 200 mL MRS broth) or 2 mM gluconasturtiin from *B. verna* DSM extracts (2.9 mL aqueous extract in 200 mL MRS broth). When the cultures reached a concentration of 10^8^ CFU/mL, enriched broths were immediately filtered (0.22 µm Sartolab RF/BT, Sartorius Stedim, Firenze, Italy) and stocked in sterile conditions until use. Aliquots of 100 mL of the *L. acidophilus* suspensions were centrifuged and supernatant, with or without GSL enriched extracts, were used for experiments. Lactic acid bacteria (LAB) broths obtained by lactobacilli fermentation alone (A1) or in presence of *E. sativa* (A2) or *B. verna* (A3) were used for further experiments with dilution range 1:2–1:100 (*v*/*v*).

### 2.4. Pathogenic Bacteria Strain and Culture Conditions

The EHEC serotype O157:H7 strain (ATCC 700728), and *Staphylococcus aureus* (ATCC 25293) were obtained from the American Type Culture Collection. Strains were routinely cultured in Columbia agar with 5% sheep blood and before experiment they were grown overnight in MH broth on the shaker (200 rpm) at 37 °C in aerobic condition. The optical density (OD) of the suspensions was measured at 600 nm (OD_600_), and bacterial cultures were diluted before use to obtain the selected working concentrations.

### 2.5. Caco-2 Cell Culture

The human colon adenocarcinoma cell line Caco-2 (ATCC HTB-37) were grown in DMEM high glucose, 10% heat-inactivated FBS, 1% NEAA, penicillin and streptomycin at 37 °C in an atmosphere of 5% CO_2_. Prior to experimental trials with EHEC, cell culture medium was changed to an antibiotic-free medium. Cells were routinely maintained in 25 cm^2^ tissue-culture treated flaks. For experiments on unpolarized monolayer, cells (passages 30–35) were trypsinised and seeded onto tissue culture treated plates (6-well, 24-well and 96-well plate depending on assays) and media were changed every 2–3 days. For studies on differentiated cells, Caco-2 were seeded onto 24-mm diameter Transwell filter unit with a 0.33 cm^2^ porous filter membrane (0.2 μm pores) and the media (0.5 mL in both apical and basolateral compartments) were replaced every other day. Cells were used for experiments following 14–16 days of culture, until a TEER (Trans-Epithelial Electrical Resistance) indicative value > 300 Ω × cm^2^ was achieved.

### 2.6. Cell Viability Bioassay

The cell viability of the broth solutions towards the human intestinal cells was evaluated by WST8 (2-(2-methoxy-4-nitrophenyl)-3-(4-nitrophenyl)-5-(2,4-disulfophenyl)-2H-tetrazolium, monosodium salt) that, in the presence of an electron mediator, is reduced, by dehydrogenases in cells (as a vitality biomarker), to formazan dye which is soluble in the tissue culture medium. The amount of formazan dye generated by dehydrogenases in cells is directly proportional to the number of living cells [39]. For experiment, Caco-2 cells were seeded in a transparent 96-well plate at a density of 5 × 10^3^ cells/well. After 80% confluence had been reached, cells were treated with LAB broths obtained by lactobacilli fermentation alone (A1) or in presence of *E. sativa* (A2) or *B. verna* (A3) (dilution 1:2; 1:10; 1:100) in complete culture medium for 24 h. As positive control, LPS were used at a concentration of 1 μg/mL. The decrease in absorbance between the treatment after 24 h (representing t_1_) and the control (representing t_0_) was monitored at 37 °C at OD_450_ using a Varioskan™ Flash Multimode Reader.

### 2.7. Antimicrobial Activity

The in vitro antimicrobial activity of the LAB broths was evaluated regarding EHEC and *S. aureus* ATCC 25293 by means of a standardized broth microdilution method [40]. Briefly, for antibacterial determinations, a suspension at 0.5 McFarland of each reference strain was prepared from an overnight culture, diluted 1:200 in MH broth and incubated with dilutions of the stock solutions in the range 1:2–1:100 (*v/v*). A number of wells were reserved in each microplate for negative (no inoculum added) and positive growth controls. The microplate was incubated at 37 °C for 24 h, and subsequently the OD_630_ was measured and used to determine microbial growth percentage values relative to the positive control.

### 2.8. Infection of Caco-2 Cells with EHEC

Caco-2 cells were grown on 6-well tissue culture plates (~ 3 × 10^6^/well) with an antibiotic-free medium for 24 h before bacterial inoculation. Thereafter, monolayers were washed twice with warm PBS and infected with an overnight culture of either EHEC at different multiplicity of infection (MOI 1–100) or *S. aureus* (MOI 100) for 2 h, 4 h and 8 h at 37 °C and 5% CO_2_. At each time point, monolayers were stained in situ with trypan blue (0.2% in PBS) for the assessment of cell membrane integrity and imaged by using a light microscope.

EHEC infections were also carried out on Caco-2 polarized monolayers grown on the top surface of the porous Transwell membrane. For this purpose, Caco-2 cells were seeded at a density of 1 × 10^5^ cells/well on the Transwell unit for 14–16 days in DMEM supplemented with 10% (*v/v*) FBS, 1% (*v/v*) NEAA and 1% (*v/v*) P/S. Both the apical and basolateral media was changed every other day. TEER readings were taken at regular intervals during the experimental time course with the Electrical Resistance System, Millicell ERS-2 (Millipore), according to the manufacturer’s instructions. Experiments were carried out after the epithelial monolayer became polarized, i.e., when TEER values ranged between 300 and 350 Ω × cm^2^ [41]. For infection (MOI 100), EHEC were added to the apical chamber after having replaced an antibiotic-free media in both chambers 24 h prior to infection. At different time points after bacterial inoculation, TEER was measured across the Caco-2 monolayer in Transwell inserts. In addition, TEER readings were obtained for Caco-2 monolayers pretreated with the LAB broths A1, A2 and A3 in order to assess their effects on the integrity of the intestinal barrier after EHEC infection. For the analysis of the anti-inflammatory properties of the LAB broths, polarized Caco-2 monolayers were grown for 24 h in antibiotic-free media containing bacterial broths A1, A2 and A3 (dilution 1:10), then EHEC infected for 2 h at 100 MOI and CXCL8 expression, as a marker of inflammation, quantitatively evaluated by means of Real Time PCR assays and ELISA (detailed protocols are described below).

### 2.9. Immunofluorescence for the Tight Junction-Associated Protein Zona Occludens-1 (ZO-1)

Immediately following TEER determinations, cell monolayers were processed for indirect immunofluorescence microscopy to detect ZO-1. Monolayers were fixed with 4% paraformaldehyde in PBS for 10 min at room temperature, washed with PBS and blocked with PBS-BSA 5% in the apical compartment for 2 h at room temperature. Then, monolayers were incubated with anti-ZO-1 antibody diluted 1:100 in PBS-BSA 5% overnight at 4 °C. The monolayers were rinsed in PBS before the incubation with the DyLight488 conjugated anti-rabbit IgG, diluted 1:200 in PBS-BSA 5% for 1 h at room temperature. After additional washings, the polycarbonate membrane was cut out from the Transwell support, mounted on a glass slide, and observed using a Nikon Eclipse E400 fluorescence microscope with DS–Fi1 digital camera.

### 2.10. RNA Extraction and Quantitative Real Time PCR

Caco-2 cells were incubated with either EHEC at different multiplicity of infection (MOI range of 1–100) or *S. aureu*s for 2 h, 4 h and 8 h. Total RNA was extracted using the RNeasy Mini Kit following the manufacturer’s instructions [42]. RNA concentration and purity were determined by NanoDrop 2000 spectrophotometer (Thermo Fisher Scientific, Waltham, MA, USA). 25 ng of total RNA were reverse transcribed using the SuperScript^®^ III First-Strand Synthesis SuperMix and amplified using the EXPRESS SYBR^®^ GreenER™ qPCR SuperMix according to the manufacturer’s protocol at a final volume of 20 µL. Real-time PCR reactions were conducted on a RotorGene Q Qiagen Real-Time PCR System (QIAGEN GmbH, QIAGEN Strasse 1, D-40724 Hilden), with an initial 5 min incubation at 60 °C, then 2 min at 95 °C, followed by 40 cycles of amplification: 95 °C for 15 s and 60 °C for 1 min, and examined by Rotor-Gene Real-Time Analysis Software 6.0 (QIAGEN GmbH, QIAGEN Strasse 1, D-40724 Hilden, Germany). Primer concentration was 500 nM. The following primers were used: CXCL8 forward 5′- CCACCGGAAGGAACCATCTC-3′ reverse 5′- GGCAAAACTGCACCTTCACA-3′; RPL13A forward 5′- CACCCTGGAGGAGAAGAGGA-3′, reverse 5′- CCGTAGCCTCATGAGCTGTT-3′. Changes in gene expression were calculated by the 2^−ΔΔCt^ formula using RPL13A as reference gene.

### 2.11. ELISA

Supernatants of culture media and Caco-2 cell RNA were collected from the same well. Supernatants were frozen at −20 °C for determination of the concentrations of CXCL8 by ELISA (Elabscience, US) according to the manufacturer’s instructions. Plates were read at 450 nm using a Varioskan™ Flash Multimode Reader. The amount of cytokine was quantified within each supernatant in triplicate.

### 2.12. Statistical Analysis

Results are expressed as mean ± SD of at least three independent experiments. Differences between the means were determined by unpaired student’s t-test or one-way ANOVA followed by Bonferroni multiple comparison test using the GraphPad Prism Software, version 6.0 (GraphPad Software, Inc., La Jolla, CA, USA).

## 3. Results and Discussion

### 3.1. Characterization of the Extracts

*E. sativa* and *B. verna* DSMs were characterized in % residual oil, profile and content of GSLs. The oil extraction came to 20.7% and 14.2% residual oil content in *E. sativa* and *B. verna* DSMs, respectively. The GSL content accounted for total 131 ± 3 µmoL g^−1^ glucoerucin, and 5.6 ± 0.8 µmoL g^−1^ glucoraphanin in *E. sativa* DSM, while *B. verna* DSM profile of GSLs was characterized only by gluconasturtiin at a concentration of 123 ± 3 µmoL g^−1^. Recoveries of total GSLs were > 95% in both microwave extracts as showed in Table 1.

### 3.2. Lactobacillus Acidophilus Broth’s Safety in Human Intestinal Cells

**Figure 1:**
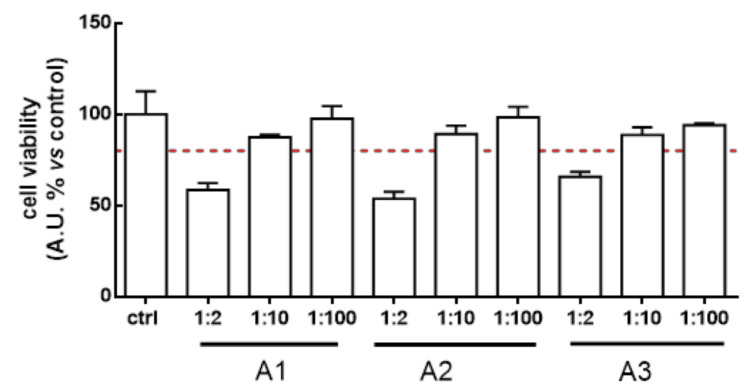
Caco-2 cells were treated with A1, A2 and A3 broths (dilution range 1:2–1:100) for 24 h. At dilution 1:2, broths showed a decrease in cell viability of less than 80%, whereas at higher dilutions the cell viability is acceptable (more than 80%).

The ability of the LAB broth to directly affect the bacterial growth of two human pathogens (EHEC and *S. aureus*) and to prevent pathogen-induced intestinal barrier dysfunction and inflammation was evaluated, having assessed their effects on Caco-2 cells. Indeed, bacterial broths obtained by lactobacilli fermentation alone (A1), in presence of *E. sativa* (A2), or *B. verna* (A3) DSM extracts, were subjected to dose-effect safety experiments in Caco-2 cells (dilution range 1:2—1:100, *v/v*). We chose low dilutions because we assumed that these conditions reflect the LAB “fermentates” quantity in the gut. At dilution 1:10 and 1:100, the samples analysed did not show a significant reduction (more than 80%) of cell viability after a 24 h treatment (Figure 1). According to these results we decided to use the 1:10 dilution for subsequent experiments.

### 3.3. Lactobacillus Acidophilus Broth Effects on Human Pathogenic Bacteria

The probiotic broths herein obtained were assayed in vitro to measure their inhibitory effect on EHEC and *S. aureus* growths. *S. aureus* and *E. coli* occur as normal flora of the skin and mucous membranes and in the gastrointestinal tract of humans, respectively; however, virulent strains, resistant to widely used antibiotics, are the most common pathogens causing healthcare-associated infections and bacteremia. The EHEC serotype O157:H7, selected in the present study, is a major foodborne pathogen which directly disrupts epithelial cell architecture and intercellular tight junctions, thus being suitable as prototype to assess the protective potential of the broth solutions. Data indicated that the broth solutions did not display inhibitory properties on the microbial growth at tested dilution range (data not shown).

### 3.4. EHEC Infection of Caco-2 Cells Modelling

Rapid resealing of the epithelial surface barrier following injuries or physiological damage is essential to control inflammation and to restore and maintain intestinal homeostasis.

**Figure 2:**
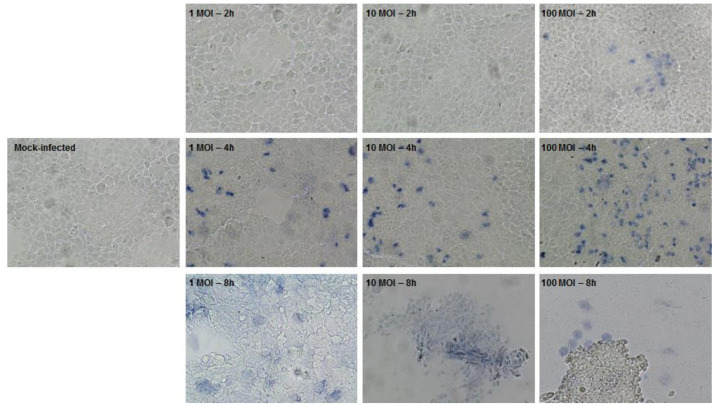
Assessment of cell membrane integrity by trypan blue exclusion staining in a time course (2–8 h) of unpolarized Caco-2 cells infection with Enterohemorrhagic Escherichia coli (EHEC) (1–100 multiplicity of infection (MOI)). Dead or damaged cells stain blue. Differences in cell monolayer morphology can be observed among the mock-infected control and cells at the different experimental conditions (10× magnification).

In the present study an accurate modelling of EHEC-Caco-2 cell interaction was carried out. Indeed, although the Caco-2 cell line is widely used in pharmacokinetic assays for drug absorption and basic research on epithelial barrier function [43], some parameters specifically related to bacterial infections have to be optimized, including the MOI and the incubation time with the selected pathogenic bacteria. Thus, cell monolayers of unpolarized Caco-2 were inoculated with different amounts of EHEC and of *S. aureus*, as control. At each time point, cells were observed and stained with trypan blue enabling the evaluation of morphological changes and cell membrane integrity as the dye penetrates into the membranes of dead cells, whereas the dye is excluded from live cells with intact cell membrane (Figure S1 and Figure 2).

Figure 2 depicts the time course of destruction of the monolayer (range 2–8 h) using different MOI (range 1–100). Few scattered cells within the monolayer stained blue at 100 MOI following 2 h post-infection, suggesting the initial cell membrane damage. Infection with EHEC led to destabilization of the monolayer within 4 h for the highest MOI, while within 8 h for the other experimental conditions. Remarkably, disruption of the monolayer integrity is supported by the increased number of blue cells correlated with the MOI at 4 h post-infection and by intense detachment of individual and groups of Caco-2 cells at 8 h post-infection.

To broadly define the biological parameters in the host pathogen interaction EHEC–Caco-2, a quantitative Real Time PCR assay measuring the CXCL8 expression, as a marker of inflammation, was carried out. CXCL8 is a potent stimulator of neutrophil activation and chemotaxis within the intestinal mucosa and its upregulation is associated with numerous acute and chronic inflammatory disorders [44]. In line with our data, an increased expression of TNF-α, CXCL1 and CXCL8 mRNA levels when Caco-2 cells were infected with EHEC has been reported [45]. CXCL8 expression can be modulated by other pro-inflammatory stimuli such as lipopolysaccharides, IL-1 or TNF-α [46]. Patients with diarrhea-associated hemolytic uremic syndrome caused by EHEC O157:H7 have high circulating levels of CXCL8. So CXCL8 has been identified as a risk factor for the pathogenesis of the disease and has been associated with high counts of polymorphonuclear leukocytes [47]. EHEC O157 bacteria, as well as single bacterial components including purified virulence proteins and O157 LPS, are capable of inducing secretion of CXCL8 from human intestinal epithelial cells [48,49,50]. Hence, the ability of EHEC to induce proinflammatory CXCL8 response in intestinal epithelial cells, which are the first cellular targets encountered by the pathogens during infection, is considered a critical step in the pathogenesis of several intestinal diseases. Herein, a confluent, differentiated and polarized layer of enterocyte-like Caco-2 cells was grown on Transwell insert as defined in the supplementary material (Figure S2). Then, polarised Caco-2 cell monolayers were EHEC infected (range 1–100 MOI) at different time points (2–4–8 h).

**Figure 3:**
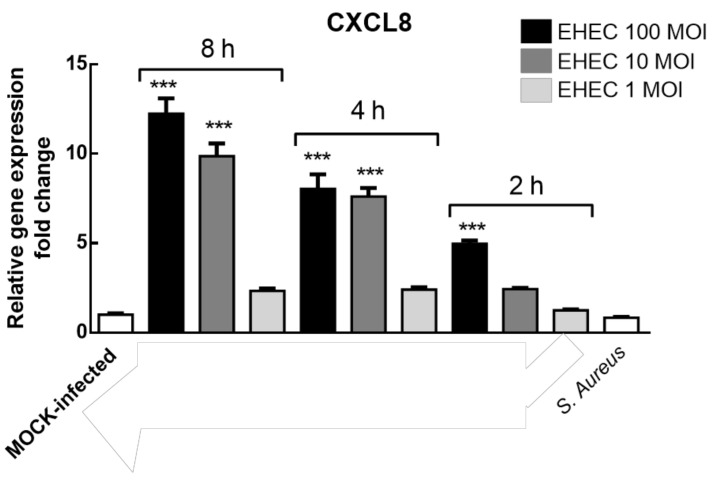
Relative interleukin-8 (CXCL8) gene expression fold change in polarized Caco-2 cell monolayers infected with EHEC at different MOI (1–100 MOI) and times post infection (2–8 h). After 2 h (at 100 MOI) and 4–8 h (at 10 and 100 MOI) of EHEC infection, CXCL8 gene expression was significantly increased (*** *p* < 0.001) in respect to the MOCK-infected cells. Caco-2 cells infected with *S. aureus* at 100 MOI for 2 h did not show a modulation of CXCL8 expression. Data were gathered from three independent experiments performed in triplicate.

Figure 3 displays the relative CXCL8 gene expression fold change in our co-culture model system; the highest levels of gene expression were measured in Caco-2 cells incubated with EHEC at 10 and 100 MOI for 4–8 h (*p* < 0.001) and with 100 only MOI for 2 h (*p* < 0.001), while at 1 MOI no statistically significant difference with the control (mock-infected Caco-2 cells) was detected at each time point. To determine the strain-specificity of EHEC infection in CXCL8 induction, we infected polarized Caco-2 cells with *S. aureus* (100 MOI for 2 h) and any significant effect was detected, suggesting that CXCL8 expression is, at least in part, pathogenic strain specific.

### 3.5. Effects of Probiotic Metabolites on CXCL8 Production by Polarized Caco-2 Cells Infected with EHEC

Caco-2 cells incubated with EHEC display an activation of defence response genes associated with oxidative stress and inflammation [51,52]. Indeed, in the bacteria co-culture system, the host cells experience an altered environment compared with the germ-free system that includes reduced pH, depletion of major energy substrates, and accumulation of fermentation by-products. Measurement of intracellular Caco-2 cell metabolites revealed significantly increased lactate, as well as changes in tricarboxylic acids (TCA) cycle intermediates [51].

In order to assess the specific anti-inflammatory properties of the probiotic strains in the gut, CXCL8 production was evaluated by incubating polarized Caco-2 cell monolayers, pretreated with lactobacilli broths, challenged or not with EHEC at 100 MOI for 2 h.

**Figure 4:**
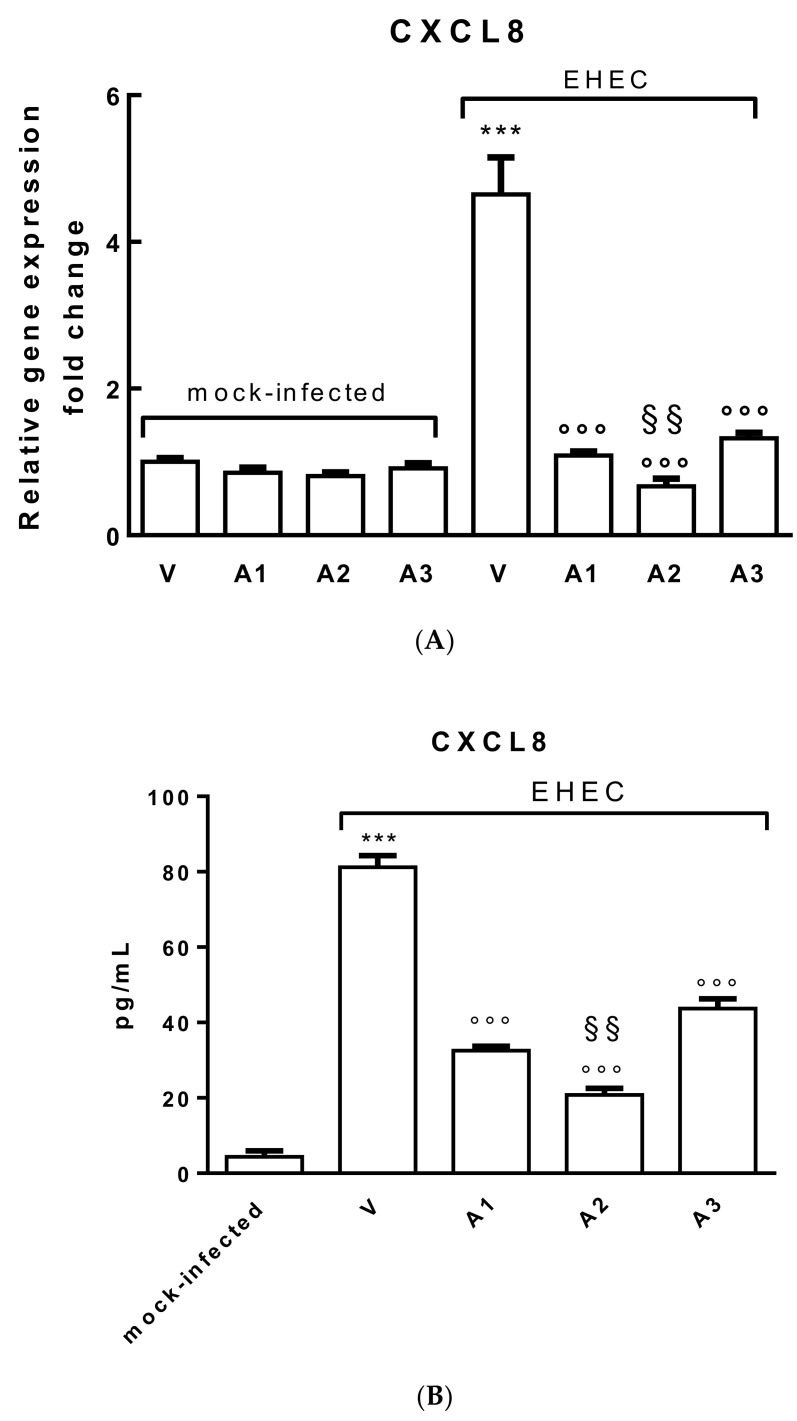
(**A**) Relative CXCL8 gene expression increased in polarized Caco-2 cell monolayers treated with the broth solutions or the vehicle (V, MRS broth) for 24 h and/or infected with EHEC at 100 MOI for 2 h (*** *p* < 0.001). A1, A2 and A3 (1:10 dilution) pretreatment significantly decreased CXCL8 expression (°°° *p* < 0.001) in respect to cells treated with EHEC; A2 shows a greater effect compared with A1 and A3 (^§§^ *p* < 0.01). (**B**) Basolateral secretion of CXCL8 by polarized Caco-2 cell monolayers after 2 h of incubation with EHEC alone (V) at 100 MOI or pretreated with A1, A2 and A3 (1:10 dilution) for 24 h. The concentration of proinflammatory mediator CXCL8 was determined by ELISA assay (in pg/mL). Mock-infected Caco-2 cell monolayers (no bacteria) served as a control. In mock-infected cells, CXCL8 is almost undetectable; after 2 h of EHEC infection at 100 MOI, CXCL8 protein significantly increased (*** *p* < 0.001). A1, A2 and A3 (1:10 dilution) pre-treatments significantly decreased CXCL8 expression (°°° *p* < 0.001) in respect to cells treated with EHEC; A2 showed a greater effect compared with A1 and A3 (^§§^ *p* < 0.01). Data were gathered from three independent experiments performed in triplicate.

As shown in Figure 4A, pretreatment with A1, A2 and A3 lactobacilli broths (dilution 1:10) induced a significant decrease in CXCL8 expression (*p* < 0.001) in respect to the vehicle (V, MRS broth), in polarized Caco-2 cells infected with EHEC (100 MOI for 2 h). Interestingly, treatment with A2 significantly decreased CXCL8 expression with respect to A1 and A3 treatments (*p* < 0.01), suggesting a synergistic role of pre-treatment with *E. sativa* seed extract. In Figure 4B, CXCL8 protein expression is shown and data confirms previous results.

Moreover, A1, A2 and A3 pretreatment upregulated the anti-inflammatory cytokine transforming growth factor β-1 (TGFβ-1) expression compared with untreated, infected cells; in line with previous data, A2 exerted a significant increase in TGFβ-1 expression compared with A1 and A3 treated infected cells (*p* < 0.01) (Figure S3).

As reported above, functionally unbalanced and uncontrolled expression of pro-inflammatory cytokines compromises intestinal integrity and promotes disease progression. Indeed, G31P, a mutant form of CXCL8 protein (CXCL8(3-72)K11R/G31P) able to inhibit CXCR1, reducing endogenous CXCL8 expression, exhibits therapeutic potential against IBD, particularly ulcerative colitis (UC), and seems to demonstrate selective synergistic effect when administered with *Lactobacillus acidophilus*, reducing also the expression of other inflammatory cyto- and chemokines (such as TNF-α, IFN-γ, IL-1β, IL-6) [53].

*L. plantarum* revealed some anti-inflammatory activity through inhibition of CXCL8 expression and increase of the anti-inflammatory cytokine IL-10 in Caco-2 cells [54,55] while a combination of selected LAB (*L. plantarum* DU1, *L. farciminis*, *Weissella cibaria* DU1, and *L. pentosus*) reduces hepatic steatosis and attenuates the inflammatory response in vitro through IL-6, CXCL8, CCL2, and TNF-α inhibition via modulation of Toll-like Receptor (TLR) negative regulators of the MAPK and NF-κB pathways [56]. Ex vivo studies in ileal and colonic mucosa from 10 post-infectious irritable bowel syndrome patients (PI-IBS), and 10 healthy controls pretreated with *L. casei* DG (LC-DG) showed that LC-DG significantly reduced pro-inflammatory cytokines IL-1α, IL-6 and IL-8 mRNA levels as well as TLR-4 protein expression after lipopolysaccharide (LPS) stimulation [57].

*E. sativa* seed extract contains several compounds with antioxidant activity, including GSLs, flavonoids (quercetin, kaempferol and isorhamnetin), carotenoids and vitamin C [58]. Furthermore, the presence of *E. sativa* in culture broth of *L. acidophilus* increased the antioxidant activity of the medium in comparison to control broths, as well as the bacteria’s own antioxidant power and antimicrobial activity [20]. To our knowledge, this is the first evidence of a significant anti-inflammatory effect of bacterial broths obtained by *L. acidophilus* fermentation in the presence of *E. sativa* seed extract, triggered by a decrease of CXCL8 expression.

### 3.6. Functional Effects of LAB Broths on Barrier Integrity in Polarized Caco-2 Cells Infected with EHEC

The epithelial surface barrier is crucial in separating a broad spectrum of noxious and immunogenic substances present in the lumen of the gut from the underlying mucosal immune compartment, as direct interaction would result in frank and uncontrolled intestinal inflammation and general immune responses [59]. When *E. coli* adheres to cells lining the intestine, disruption of the barrier function occurs leading to increased cell permeability through recruitment of pro-inflammatory cytokines such as CXCL8 and TNF-α. These inflammatory cytokines modulate several signalling pathways within the host that promote and redistribute tight junction proteins, which increases membrane permeability and paracellular movement of bacteria. Previous studies have suggested that at least in part through CXCL8 inhibition *Lactobacillus plantarum* L9 and *Lactobacillus acidophilus* LA have good capacities to adhere to the enterocyte monolayer surface of Caco-2 cells resulting in significant inhibition of *E. coli* adhesion and cell internalisation [55].

**Figure 5:**
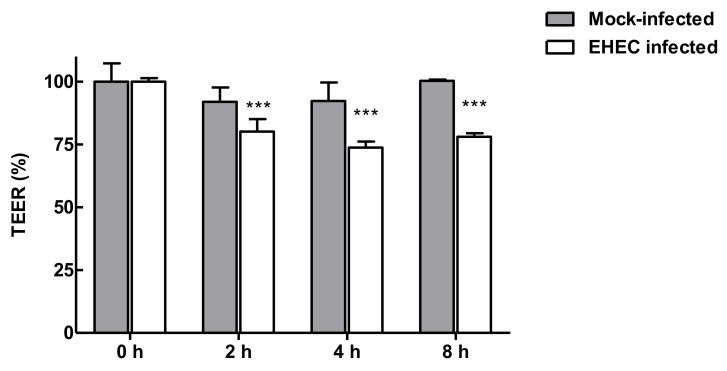
Trans-Epithelial Electrical Resistance (TEER) values of Caco-2 cell monolayer cultures inoculated with EHEC at 100 MOI over time in comparison to mock-infected control (*** *p* < 0.001).

Herein, a confluent, differentiated and polarized layer of enterocyte-like Caco-2 cells was grown on Transwell insert and, once optimized (Figure S2), the model system was used to evaluate the properties of LAB broths in preventing epithelial injury following EHEC infection. In detail, polarised Caco-2, with TEER values between 300–350 Ω × cm^2^ (approximately two weeks after the seeding), were EHEC infected (MOI 100) and the disruption of the barrier was monitored at various intervals. At 2 h post-infection, the TEER measurement was significantly reduced compared to the corresponding mock-infected sample, which indicated an alteration of membrane integrity, thus this time point was selected for subsequent experiments (Figure 5).

**Figure 6:**
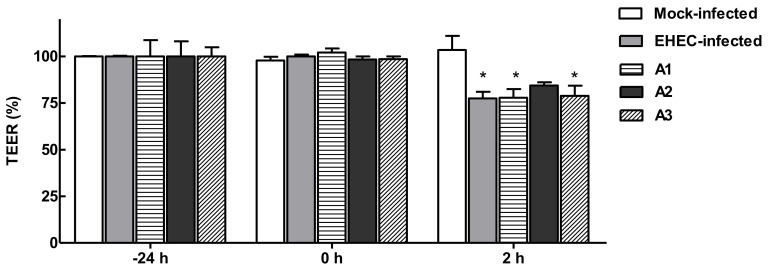
TEER values of Caco-2 monolayers at different experimental conditions: before experiments (−24 h), following a 24 h of treatment with A1, A2, A3 (0 h) and at 2 h post infection with EHEC at 100 MOI (2 h). TEER values significantly decreased when Caco-2 cells were untreated and pretreated with A1 and A3, thereafter EHEC infected (* *p* < 0.01).

In order to monitor the ability of the LAB broths A1, A2 and A3 to prevent barrier dysfunction, the TEER of Caco-2 cells infected with EHEC was measured in their absence and presence (Figure 6). In particular, different experimental conditions were used and data were compared. The differentiated intestinal cells grown onto Transwell insert were incubated before bacterial inoculation, with tenfold dilution of broth solutions. Samples were added in both medium chambers, and TEER values were measured. As readings remained stable over this incubation, the safety of the samples on Caco-2 cells is assumed, also in accordance with cell viability results. Thereafter, pathogenic bacteria were apically added (MOI 100) and TEER values were assessed after 2 h. As controls, untreated/mock-infected Caco-2 cells and untreated/infected Caco-2 cells were used in each experimental testing.

**Figure 7:**
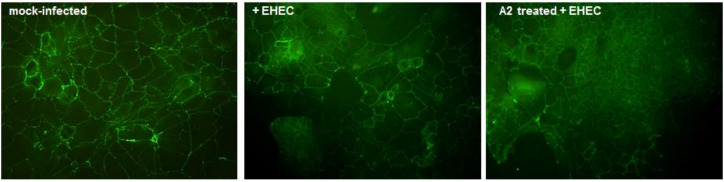
Distribution of zona occludens-1 (ZO-1) in Caco-2 monolayers at different experimental conditions. Mock-infected cells show a well-circumscribed tight junction bands over the entire image field. EHEC infected cells (100 MOI for 2 h) display multiple areas of junctional disruption without visually appreciable differences when pretreated with A2 (10× magnification).

As depicted in Figure 6, TEER values of EHEC-infected Caco-2 cells significantly decreased by 22.5% compared to mock-infected cells (*p* < 0.001) indicating a membrane-damaging effect. TEER readings of infected cells pretreated with LAB broths (1:10 dilution) also decreased, suggesting the disruption of the tight junctions of Caco-2. Interestingly, TEER values of Caco-2 cells pretreated with A2 broth and thereafter infected were no significantly different from mock-infected cells when Bonferroni’s Multiple Comparison Test was used to compare data; this result suggests that lactobacilli fermented broth enriched with *E. sativa* extract reduced the pathogen-induced changes in barrier integrity. Having demonstrated that A2 broth did not exert antimicrobial activity on EHEC, it is possible to speculate that A2 is an effective in vitro inhibitor of epithelial injury caused by EHEC acting on the intestinal barrier, possibly through its anti-inflammatory activity. Caco-2 monolayers immunostained for tight junction protein ZO-1 displayed a uniform intercellular architecture in the control monolayer (mock-infected) while Caco-2 infected with EHEC revealed a more diffuse staining with multiple areas of complete barrier disruption. Treatment with A2 prior to pathogen infection did not remarkably prevent EHEC redistribution of ZO-1 staining, confirming its modest positive effect on the intestinal barrier (Figure 7).

A2 is the product of *L. acidophilus* fermentation of standard MRS broth enriched with *E. sativa* extract diluted in order to achieve 2 mM glucoerucin. Glucoerucin is the precursor of the ITC erucin of which sulforaphane is an oxidized metabolite [37]. Sulforaphane protects and repairs the injury to the mucosal epithelium of the colon and cecum in mice with chemical-induced bladder cancer, through the decreasing of inflammation, with a reduction in the release of cytokines (IL-6); and protects immune response, with a reduction of secretory immunoglobulin A [60]. Despite the limited studies of erucin in comparison to those of sulforaphane, beneficial properties of erucin similar to sulforaphane have recently been reported together with its ability to release H_2_S in vitro and to mediate vasodilatation [37,61]. Natural ITCs, such as erucin, or allyl ITC, highly present in black mustard (*Brassica nigra* L.), 4-hydroxybenzyl ITC, highly present in white mustard (*Sinapis alba* L.), and benzyl ITC, highly present in garden cress (*Lepidium sativum* L.) have been described as slow H_2_S-releasing compounds [62]. H_2_S is a well-known endogenous gas transmitter that plays pivotal roles in the cardiovascular system [61], and in regulating cell growth [23]; moreover, in the gastrointestinal tract H_2_S has been shown to reduce inflammation and accelerate healing of damaged tissue (such as ulcers), while suppression of H_2_S production results in impaired healing of tissue injury and exacerbation of inflammation [63,64].

## 4. Conclusions

The present study examined the biological properties of three bacterial broths obtained by lactobacilli fermentation alone (A1), in presence of *E. sativa* (A2), or *B. verna* (A3). The broth obtained by *L. acidophilus* fermentation in the presence of enriched extracts from *E. sativa* seeds, characterized by a high concentration of glucoerucin, induced a significant decrease in the CXCL8 expression in Caco-2 cells following EHEC infection, and reduced epithelial disruption caused by EHEC when intestinal cells were cultured to form a fully differentiate, confluent and tight monolayer.

To our knowledge this is the first study employing complementary in vitro models of gut injury to study the effects of “functionalised” probiotics in the presence of enteric bacterial pathogens. The findings of the current study demonstrate that pre-treatment with *E. sativa* seed extract may increase the beneficial effect of Lactobacilli in EHEC-injured gut.

This supplemented fermentation, which successfully enhances the protective effects against gut inflammation and barrier dysfunction induced by pathogens, provides the foundation for the further development and application of this kind of fermentation in food industry. Indeed, lactic acid bacteria are widely used as starter cultures for the production of diverse fermented foods but, to the best of our knowledge, the protective role of their fermented broths in gastrointestinal disorders has not been extensively studied.

Even if these results are very promising, in the near future a pilot trial in human subjects will be necessary to determine whether “super probiotics” administration contributes to the maintenance and protection of intestinal microflora during enteric infections.

## Figures and Tables

**Table 1:** Recovery % of total glucosinolates (GSLs) from *Eruca sativa* and *Barbarea verna* extracts obtained, starting from 30 g of defatted seed meals, as evaluated by HPLC–UV analysis of desulpho-glucosinolates. Glucosinolate content expressed as mean ± standard deviation of three replicates.

Exctract	Final Volume (mL)	Total GSLs (µmoL mL^−1^)	Recovery (%)
***Eruca sativa***	20	201 ± 5	98
***Barbarea verna***	26	142 ± 4	100

## Supplementary Materials

The following are available online at https://www.mdpi.com/2072-6643/12/10/3064/s1, Figure S1: Time course (2–8 h) of destruction of Caco-2 monolayer following EHEC infection at different MOI (100-1); Figure S2: Optimization ofCaco-2 cell culture in a Transwell system device; Figure S3: Relative TGFβ-1 gene expression in polarized Caco-2 cell monolayers treated for 24 h with the broth solutions (or the vehicle, V, MRS broth) and infected with EHEC at 100 MOI for 2 h.
