# Supplementary material for: Effect of Lactobacillus acidophilus Fermented Broths Enriched with Eruca sativa Seed Extracts on Intestinal Barrier and Inflammation in a Co-Culture System of an Enterohemorrhagic Escherichia coli and Human Intestinal Cells
Source: Nutrients. 2020 Oct 7;12(10):3064. doi: 10.3390/nu12103064 (PMC7600469; doi:10.3390/nu12103064)
Supplement: Supplementary file 1 [file nutrients-12-03064-s001.pdf]

Supplementary material

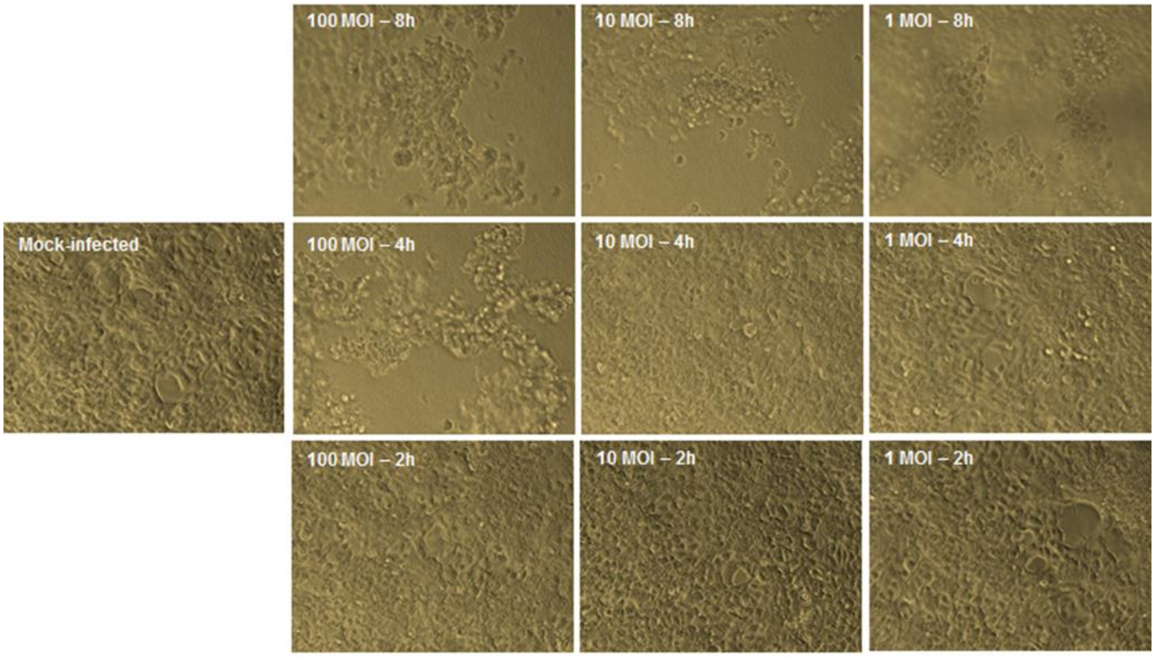

Figure S1. Time course (2-8 hours) of destruction of Caco-2 monolayer following EHEC infection at different MOI (100-1). Visual differences in cell monolayer integrity can be observed among the mock-infected control and cells at the different experimental conditions

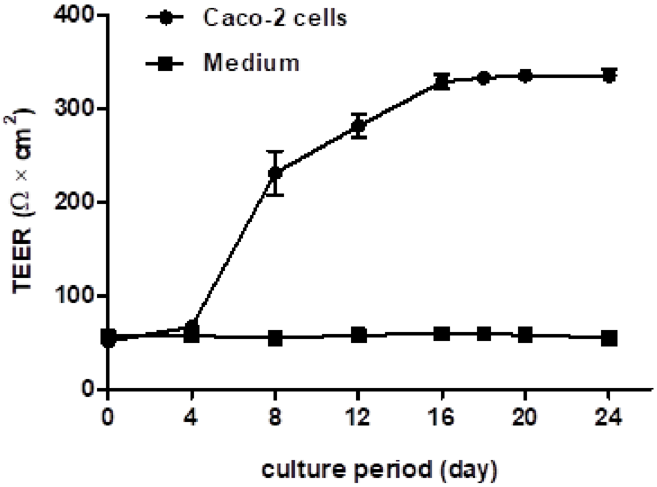

Figure S2. Optimization of Caco-2 cell culture in a Transwell system device. Cells (passages 30-35) were seeded on the Transwell membrane and TEER values were measured as a function of culture time; a continuous increase in TEER values was observed in the first 15 days thereafter reaching a plateau. Values were over 250 $\Omega \times cm^2$ that is considered as a threshold value for a standard polarized Caco-2 monolayer.

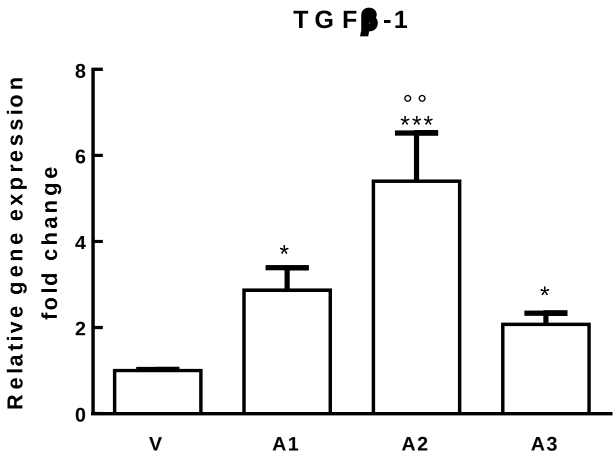

Figure S3. Relative TGFβ-1 gene expression in polarized Caco-2 cell monolayers treated for 24 hours with the broth solutions (or the vehicle, V, MRS broth) and infected with EHEC at 100 MOI for 2 h. A1, A2 and A3 (1:10 dilution) pretreatment significantly increase TGFβ-1 expression (*** $P < 0.001$; * $P < 0.05$) in respect with cells treated with just EHEC; A2 with a greater effect compared with A1 and A3 ($^{\circ\circ}$ $P < 0.01$).

The following primers were used to perform Real-Time PCR: TGFβ-1 forward 5′-CAACTATTGCTTCAGCTCCACG -3′ reverse 5′- AAGTTGGCATGGTAGCCCTT -3′; RPL13A forward 5′-CACCCTGGAGGAGAAGAGGA-3′, reverse 5′- CCGTAGCCTCATGAGCTGTT-3′. Changes in gene expression were calculated by the $2^{-\Delta\Delta Ct}$ formula using RPL13A as reference gene. Data are the pool of three independent experiments.
